# The nuclear-encoded plastid ribosomal protein L18s are essential for plant development

**DOI:** 10.3389/fpls.2022.949897

**Published:** 2022-09-23

**Authors:** Shujing Chen, Xinhuang Zeng, Yiqi Li, Shijun Qiu, Xiaoqun Peng, Xinjue Xie, Yujie Liu, Chancan Liao, Xiaoyan Tang, Jianxin Wu

**Affiliations:** ^1^Guangdong Provincial Key Laboratory of Biotechnology for Plant Development, School of Life Sciences, South China Normal University, Guangzhou, China; ^2^Guangdong Laboratory for Lingnan Modern Agriculture, Guangdong, China; ^3^Shenzhen Institute of Molecular Crop Design, Shenzhen, China

**Keywords:** plastid ribosomal protein, chloroplast, albino seedling, Arabidopsis, rice, intron splicing, embryo development

## Abstract

Plastid ribosomal proteins (PRPs) are necessary components for plastid ribosome biogenesis, playing essential roles in plastid development. The ribosomal protein L18 involved in the assemble of 5S rRNA and 23S rRNA, is vital for *E. coli* viability, but the functions of its homologs in plant plastid remain elusive. Here, we characterized the functions of the plant plastid ribosomal protein L18s (PRPL18s) in Arabidopsis and rice. *AtPRPL18* was ubiquitously expressed in most of the plant tissues, but with higher expression levels in seedling shoots, leaves, and flowers. AtPRPL18 was localized in chloroplast. Genetic and cytological analyses revealed that a loss of function of *AtPRPL18* resulted in embryo development arrest at globular stage. However, overexpression of *AtPRPL18* did not show any visible phenotypical changes in Arabidopsis. The rice OsPRPL18 was localized in chloroplast. In contrast to *AtPRPL18*, knockout of *OsPRPL18* did not affect embryo development, but led to an albino lethal phenotype at the seedling stage. Cytological analyses showed that chloroplast development was impaired in the *osprpl18-1* mutant. Moreover, a loss-function of OsPRPL18 led to defects in plastid ribosome biogenesis and a serious reduction in the efficiency of plastid intron splicing. In all, these results suggested that PRPL18s play critical roles in plastid ribosome biogenesis, plastid intron splicing, and chloroplast development, and are essential for plant survival.

## Introduction

Plastid is a semiautonomous organelle that possesses its own genome and protein synthesis apparatus (Zoschke and Bock, 2018). The plastid ribosome is responsible for the synthesis of plastid genome-encoded proteins, playing a pivotal role in plastid biogenesis (Tiller and Bock, 2014). Similar to prokaryotic ribosomes, the plastid 70S ribosome consists of a 50S large subunit and a 30S small subunit. The 50S subunit contains 23S rRNA, 5S rRNA, 4.5S rRNA, and 33 ribosomal proteins, while the 30S subunit contains 16S rRNA, and 24 ribosomal proteins (Tiller and Bock, 2014). Interestingly, 36 out of the 57 ribosomal proteins are encoded by nuclear genes, and the others are encoded by plastid genes (Tiller and Bock, 2014), indicating that the biogenesis of plastid translational apparatus may be tightly regulated under the cooperation of plastid and nucleus.

In the past two decades, lots of researches have shed light on the functions of plastid ribosomal proteins (PRPs). In Arabidopsis, many PRPs were confirmed to be essential for plant viability, such as AtPRPL1, AtPRPL4, AtPRPL6, AtPRPL10, AtPRPL13, AtPRPL18, AtPRPL21, AtPRPL27, AtPRPL28, AtPRPL31, AtPRPL35, AtPRPS13, and AtPRPS20 (Tzafrir et al., 2004; Bryant et al., 2011; Romani et al., 2012; Yin et al., 2012). Indeed, AtPRPL1, AtPRPL4, AtPRPL21, AtPRPL27, AtPRPL35 and AtPRPS20 were vital for the transition from the globular to the heart stage of embryogenesis (Romani et al., 2012; Yin et al., 2012), and AtPRPL28 was essential for plant development during the embryo greening process (Romani et al., 2012). Some PRPs appeared to be dispensable for plant viability, but play critical roles in plant development, such as AtPRPL11, AtPRPL21, AtPRPL24, AtPRPS1, AtPRPS5, AtPSRP3, AtPSRP4, and AtPSRP5, which showed pale-green leaves and retarded plant growth (Pesaresi et al., 2001; Morita-Yamamuro et al., 2004; Romani et al., 2012; Tiller et al., 2012; Zhang et al., 2016). In contrast to Arabidopsis, a few of plastid ribosomal genes were characterized in rice. A loss function of either of the plastid ribosomal genes *OsPRPS1/ASL4*, *OsPRPS20/ASL1*, *OsPRPL12/AL1*, or *OsPRPL21/ASL2* resulted in an albino seedling death phenotype (Gong et al., 2013; Lin et al., 2015; Zhao et al., 2016; Zhou et al., 2021). The mutant with a substitution from glycine to valine (G92V) of OsPRPS9/WGL2 showed an albino phenotype at the early seedling stage, and then gradually transitioned to green (Qiu et al., 2018). However, the knock-out mutant of *OsPRPS9/WGL2* exhibited albino lethal phenotype at seedling stage (Qiu et al., 2018). Interestingly, a single amino acid residue mutation from Threonine to Isoleucine (T81I) of OsPRPL13/WLP1 led to an albino phenotype at 23°C, but no significant differences compared with wild-type plants at 30°C, while *OsPRPL13* RNAi plants displayed albino lethal phenotype (Song et al., 2014). These results suggested that PRPs are crucial for plant development.

In prokaryotic organisms, the ribosomal protein L18 is essential for the assembly of 5S rRNA and 23S rRNA. The C-terminal of L18 contains a 5S rRNA binding site, and its N-terminal is involved in the interaction of 5S rRNA and 23S rRNA (Brosius et al., 1975; Newberry and Garrett, 1980). In Arabidopsis, eight homologs of L18 were identified, including μL18-L1, μL18m, μL18c/AtPRPL18 (Plastid Ribosomal Protein L18), μL18-L4, μL18-L5, μL18-L6, μL18-L7, and μL18-L8 (Wang et al., 2020). μL18 m was confirmed to be a component of mitochondria ribosome (Waltz et al., 2019), while μL18c/AtPRPL18 was proposed to be a plastid ribosomal protein (Wang et al., 2020). A screening of nuclear genes encoding chloroplast-localized proteins required for embryo development identified a mutant (*emb3105*) of *AtPRPL18*, which displayed an unconfirmed embryo-defective phenotype in Arabidopsis (Bryant et al., 2011). However, direct evidence of the role of AtPRPL18 in plant embryo development is still lacking, and the functions of PRPL18s in other plant species remains elusive.

In the present study, a comparison of the functions of PRPL18s in Arabidopsis and rice was performed. Both of *AtPRPL18* and *OsPRPL18* were expressed highly in green tissues, and their proteins were localized in chloroplast. However, a loss function of *AtPRPL18* resulted in embryo development arrest at globular stage, while knockout of *OsPRPL18* did not affect embryo development, but chloroplast development was aborted, leading to an albino lethal phenotype at seedling stage. Furthermore, OsPRPL18 is required for plastid ribosome biogenesis and plastid intron splicing. Taken together, these data highlight the essential roles of PRPL18s in plant development in Arabidopsis and rice.

## Materials and methods

### Plant materials and growth conditions

*Arabidopsis thaliana* ecotype Columbia-0 (Col-0) and the japonica rice ecotype WuYunGeng (WYG) were used as the wild-types in this study. The *atprpl18/+* T-DNA insertion mutant (SAIL_415_H08) was obtained from Arabidopsis Biological Resource Center (ABRC). Arabidopsis seeds were surface sterilized by 2.5% NaClO for 8 min followed by 5 times rinsing with sterile water and stratified at 4°C for 2 days. The Arabidopsis plants were cultivated in a greenhouse at 22 ± 2°C under a 16 h light and 8 h dark cycle (Wu et al., 2015). Rice seeds were surface sterilized and germinated on MS medium supplemented with 3% sucrose and 0.7% agar in a growth chamber under a 10 h light/14 h photoperiod with 12,000 lux light intensity at 28°C. The rice seedlings at three-leaf stage were then planted in rice paddy field under natural conditions with regular care.

### Protein alignment and phylogenetic analysis

BLASTP was performed in NCBI^1^ and Phytozome^2^ using the full-length sequence of AtPRPL18 as a query. Fifteen representative homologues of AtPRPL18 from different species were retrieved including Crahi.0013s0018.1 (*Crambe hispanica*), Ciclev10022561m (*Citrus clementina*), Potri.010G005000.3 (*Populus trichocarpa*), GlymaLee.09G068200.1. (*Glycine max*), Vigun09g043700.1 (*Vigna unguiculata*), BdiBd213.1G0042200.1 (*Brachypodium distachyon*), LOC_Os03g61260.1/OsPRPL18 (*Oryza sativa*), Pahal.9G024200.1 (*Panicum hallii*), ACF85829.1 (*Zea mays*), Joasc.10G094600.1 (*Joinvillea ascendens*), Ceric.13G022200.1 (*Ceratopteris richardii*), Pp3c11_5070V3.1 (*Physcomitrium patens*), Cre01.g052100.t1.2 (*Chlamydomonas reinhardtii*), GIL60489.1 (*Volvox africanus*), and WP_069790223.1 (*Cyanobacterium*). The protein sequences were aligned using Clustal Omega^3^ with default parameters, and the results were visualized by Jalview.^4^ The phylogenetic tree was constructed using the Neighbor-Joining algorithm (1,000 replicates) in MEGA11 (Tamura et al., 2021).

### Gene expression and subcellular localization analysis

For gene expression analysis, quantitative RT-PCR (qRT-PCR) was carried out using SYBR Green fluorescence with LightCycler 96 System (Schmittgen and Livak, 2008). Various Arabidopsis tissues including 7-day-old seedlings, and roots, stems, mature leaves and inflorescence from 40-day-old plants, and siliques on the 5th and 15th day after fertilization (DAF) were collected. Rice vegetative tissues and panicles were collected from seedlings or mature plants at heading date stage. Total RNA was extracted and reverse-transcribed to cDNA. qRT-PCR was performed using specific primer pairs (Supplementary Table 1). The *AtACT2* (AT3G18780) gene and *OsUBI* (LOC_Os03g13170) gene were used as internal controls in Arabidopsis and rice, respectively.

For protein subcellular localization analysis, the CDSs of *AtPRPL18* and *OsPRPL18* were cloned into the vector *pSAT6-EYFP-N1* and *pCAMBIA1300* driven by 35S promoter (Tzfira et al., 2005). The resultant *pSAT6-AtPRPL18-EYFP* and *pSAT6-OsPRPL18-EYFP* vectors were introduced into Arabidopsis or rice protoplasts by polyethylene glycol (PEG)–calcium-mediated transformation (Chen et al., 2006; Yoo et al., 2007). The transformed protoplasts were observed with a laser confocal scanning microscope (LSM-800; Carl Zeiss) after 12 h incubation. The resultant *pCAMBIA1300-35S:AtPRPL18-GFP* and *pCAMBIA1300-35S:OsPRPL18-GFP* vectors were introduced into *Agrobacterium tumefaciens* strain EHA105. For subcellular protein localization, *Nicotiana benthamiana* (*N. benthamiana*) leaves were infiltrated with the indicated Agrobacteria as previously described (Sparkes et al., 2006). The GFP-tagged proteins were examined 2 d after infiltration using a confocal laser scanning microscope (LSM810, Carl Zeiss).

### Generation of transgenic plants

For mutant complementation, a 4,650 bp genomic DNA including the gene body of *AtPRPL18*, a 2,363 bp upstream sequence and a 956 bp downstream sequence, was cloned into the vector *pCAMBIA1300*. A *pCRISPR-AtPRPL18* plasmid for gene editing was constructed with the target sequence ACTAACCGCAGTGCGTTCTT according to a previously reported protocol (Yan et al., 2015). The overexpression construct was generated by introducing a gene expression cassette with *35S:AtPRPL18-NosR* into *pCAMBIA1300*. The resulted constructs were introduced into *Agrobacterium tumefaciens* EHA105 and then transformed into heterozygous mutant *atprpl18/+* or Columbia-0 with a floral dip method (Zhang et al., 2006).

The *osprpl18* mutant was generated by employing a CRISPR-Cas9 gene editing tool with the target sequence AGTGATTGCCAAGTCTTGCT and AGGACTTGGAATACTCGGCAGG on *OsPRPL18* (Ma et al., 2015), and an Agrobacterium-mediated rice transformation method (Toki et al., 2006).

For GUS staining analysis, the 2.4 kb upstream region of *AtPRPL18* was amplified by PCR using the primers AtPRPL18-GUS-F and AtPRPL18-GUS-R and cloned into the vector *pCAMBIA1301* with In-Fusion HD cloning kit. The resultant *proAtPRPL18-GUS* plasmid was transformed into Arabidopsis wild-type plants *via* the floral dip method (Zhang et al., 2006). The homozygous *proAtPRPL18-GUS* transgenic lines were identified and then used for GUS staining. The GUS staining solution includes 2 mM 5-bromo-4-chloro-3-indolyl glucuronide (X-Gluc), 0.1 % Triton X-100, 2 mM K_4_Fe (CN)_6_ and 2 mM K_3_Fe (CN)_6_ in 50 mM sodium phosphate buffer, pH 7.0 (Jefferson et al., 1987). The plant tissues were incubated in GUS staining solution at 37°C for 4 h then decolorized in 75% ethanol at 37°C overnight. The stained samples were photographed using a Leica DM6 microscope.

### Ovule clearing and embryo observation

Seeds of wild-type and *atprpl18/+* mutant were removed from siliques and totally cleared in Hoyer’s buffer [chloral hydrate: glycerol: water, 8:1:2 (w/v/v)] for 30 min to 8 h depending on the embryo developmental stage (Chen et al., 2015). Embryos were examined using a light microscope Carl Zeiss Axio Observer A1 with optimal differential interference contrast.

### Transmission electron microscopy analysis

For transmission electron microscopy (TEM) analysis, leaves from the 3-leaf-stage seedlings of *osprpl18-1* and wild-type were fixed and resin-embedded as previously described (Chang et al., 2016). Samples were sliced and stained with 0.5% toluidine blue for semithin sections and uranyl acetate and lead citrate for ultrathin sections. The stained semithin sections and stained ultrathin sections were photographed using a Zeiss Axio Imager A1 microscope and a transmission electron microscope (TEM) (JEOL JEM1400), respectively.

### Ribonucleic acid splicing analysis

Total RNA was extracted from the seedling shoots of WYG and *osprpl18-1* at the third-leaf stage using the RNA extraction kit (Omega Bio-tek, R6830). First strand cDNA was synthesized with random hexamers or RT primer mix using the PrimeScript II 1st strand cDNA Synthesis Kit (Takara, 6210A). For RT-PCR analyses, primers were designed situated on exons flanking with the concerned introns. For qRT-PCR, primers were designed positioned across intron/exon junctions of the indicated transcripts. Some primers used in this study were consistent with that in previous research (Lv et al., 2020).

## Results

### Phylogenetic analysis of plant plastid ribosomal protein L18 proteins

**FIGURE 1:**
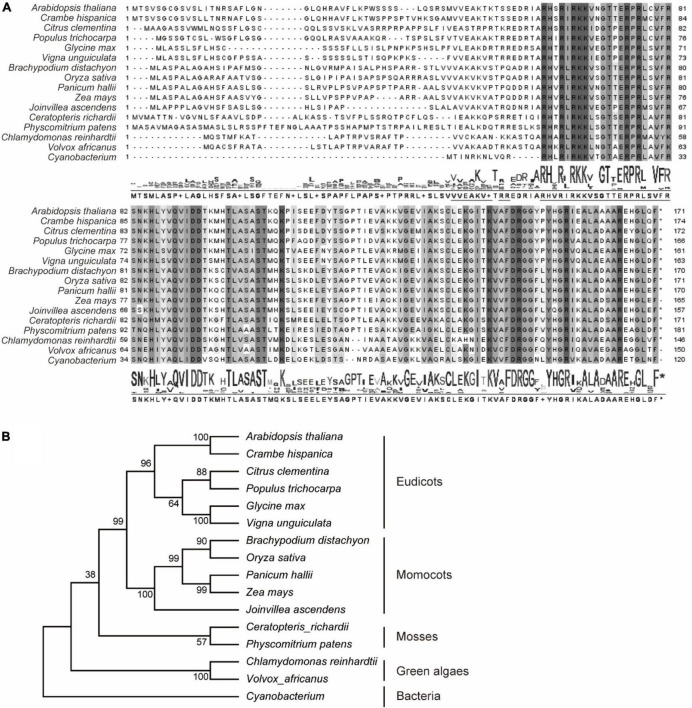
Phylogenetic analysis of RPL18s. Sixteen represented PRPL18 homologs were obtained from plants and cyanobacterium using BlastP of AtPRPL18. Multiple sequence alignment was performed using Clustal Omega **(A)**, and phylogenetic tree was constructed by MEGA11 **(B)**. The conserved amino acid residues were highlighted by gray backgrounds.

In previously reports, AtPRPL18 was identified as a plastid ribosomal protein L18 in Arabidopsis (Wang et al., 2020). To identify plant PRPL18 proteins, BlastP was employed to search homologs of AtPRPL18, and 15 L18 protein sequences from representative species were selected. Plastid is proposed originating from the engulfment of a cyanobacteria ancestor into a unicellular heterotrophic protist (Jensen and Leister, 2014). Lots of plastid genes have undergone a transfer from plastid to nucleus (Jensen and Leister, 2014). Interestingly, all of the displayed PRPL18 proteins from various eukaryotic species were encoded by nuclear genes, indicating the transfer event of PRPL18 from plastid to nucleus occurred very long time ago. However, protein sequences alignment analysis revealed that PRPL18 proteins remained high identities, especially in the protein C terminals, such as AtPRPL18 and OsPRPL18, which shared 60 and 59% identities with the full length of cyanobacterium L18 protein, respectively (Figure 1A). Phylogenetic analysis showed that AtPRPL18, together with other PRPL18 homologs of dicotyledonous plants, belong to a clan that was separated from other clans formed by monocots, mosses, green algae and prokaryotes (Figure 1B), indicating PRPL18s may retain a conserved function from their ancestor and also evolve species-specific functions.

### Expression pattern of *AtPRPL18* and subcellular localization of AtPRPL18

**FIGURE 2:**
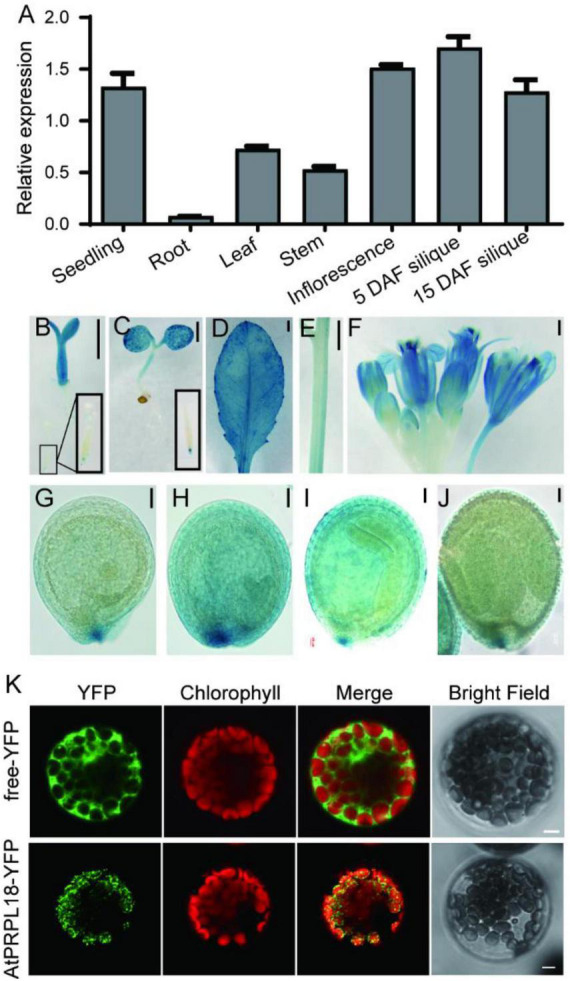
Gene expression patterns and protein subcellular localization of AtPRPL18. **(A)** The relative expression of *AtPRPL18* in seedling, and stem, mature leaf, inflorescence and siliques from 40-old-day Arabidopsis plants. *AtACT2* served as the internal control. Data represent means ± SD (*n* = 4). **(B–J)** GUS staining in the tissues of *proAtPRPL18:GUS* plants. **(B)** 3 day-old seedling, **(C)** 6 day-old seedling, **(D)** leaf, **(E)** stem, **(F)** inflorescence, **(G)** seeds at globular stage, **(H)** seeds at heart stage, **(I)** seeds at torpedo stage, **(J)** seeds with well-developed embryo. **(K)** Subcellular localization of AtPRPL18-YFP in Arabidopsis mesophyll protoplast. Free YFP was used as a control. The chlorophyll autofluorescence was used as the marker of chloroplast. Scale bars = 1 mm (B-F), 50 μm **(G–J)**, and 5 μm **(K)**.

Gene function is closely related to its expression profiles and protein localization. qRT-PCR analysis showed that the transcripts of *AtPRPL18* were more abundant in seedlings, stem, leaves, inflorescence and siliques than that in roots (Figure 2A). Moreover, GUS reporter assay was employed to further analyze the expression pattern of *AtPRPL18*. A 2,452 bp fragment upstream of the translational initiation site was fused to the GUS reporter gene and introduced into wild-type plants to generate *pAtPRPL18-GUS* transgenic plants. Consistent with the qRT-PCR results, strong GUS activities were detected in seedling shoots, leaves, flowers and young siliques, whereas in roots, only root tips displayed GUS activity (Figures 2B–F). Interestingly, a higher GUS activity was observed in embryos at heart stage (Figures 2G–J). These results suggested that *AtPRPL18* may participate in the development of green tissues as well as reproductive tissues.

The Arabidopsis PRPL18 protein was predicted to contain a typical signal peptide for chloroplast subcellular localization at its N-terminus using TargetP-2.0 software (Almagro Armenteros et al., 2019). To verify the prediction, a plasmid harboring *AtPRPL18-YFP* expression cassette was introduced into Arabidopsis protoplasts to examine the subcellular localization of AtPRPL18. In the transformed cells, the YFP fluorescence appeared in small dot-like structures and, was overlapped with the chlorophyll autofluorescence (Figure 2K). Transient expression of AtPRPL18-GFP in tobacco leaves showed that AtPRPL18-GFP was unevenly distributed in chloroplast, and also presented lots of dot-like structures (Supplementary Figure 1A). Though, these results were not well consistent with the previous report that μL18c-GFP/AtPRPL18-GFP was found to be localized diffusely throughout chloroplasts in the 35S: μL18c-GFP transgenic plants, it is obvious that AtPRPL18 is a chloroplast-localized protein in Arabidopsis. The difference in the distribution of the fluorescence signals in chloroplast may be resulted from the diverse protein expression levels in different protein localization assays.

### Phenotypes of *atprpl18/+*

**FIGURE 3:**
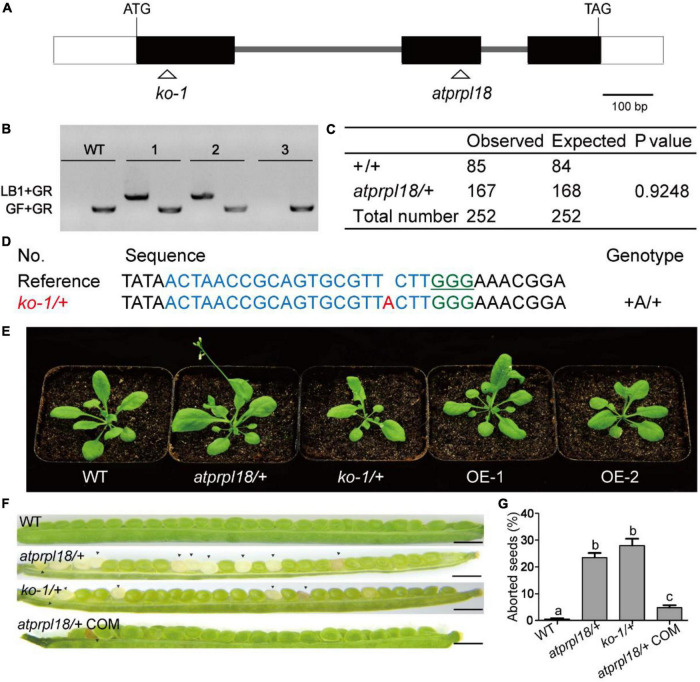
Characterization of the function of AtPRPL18 in plant development. **(A)** The gene structure of *AtPRPL18*. The gray line, white rectangle, and black rectangle represent intron, 5’/3’UTR, and exon, respectively. **(B)** PCR analysis of the genotype of the progenies of *atprpl18/+* mutant. 1, 2, and 3 were the representative progenies from *atprpl18/+* mutant. The T-DNA specific primer LB1 and genomic DNA primer GR were used to amplify T-DNA flanking sequences, GF and GR were used to amplify the genomic DNA sequence without T-DNA insertion. **(C)** Statistical analysis of the genotypes of *atprpl18/+* progenies. **(D)** Generation of *AtPRPL18* deficient mutants by CRISPR-Cas9. The guide RNA targeted DNA sequence and PAM motif were highlighted in blue and green, respectively. **(E)** 30-day-old plants of *atprpl18/+*, *ko-1*/+, and *AtPRPL18* overexpression transgenic plants OE-1 and OE-2. **(F,G)** Comparison of the seed phenotypes of WT, *atprpl18/+*, *ko-1*/+, and *atprpl18/+* COM plants. At least fourteen siliques of each genotype were examined. The rates of albino seeds in siliques were analyzed. Data represent means ± SD. Letters above bars indicate significant differences (*P* < 0.05, Student–Newman–Keuls test). This experiment was repeated twice with similar results. (a–c) Genetic analysis of the *atprpl18/+* mutant.

In order to understand the biological roles of *AtPRPL18* in Arabidopsis, a T-DNA insertion mutant (SAIL_415_H08) of *AtPRPL18* was obtained from ABRC.^5^ The T-DNA was localized in the second exon of *AtPRPL18* (Figure 3A), which was confirmed by PCR and sequencing (Figure 3B). However, no homozygous *atprpl18* mutant was identified from the progenies of *atprpl18* /+ heterozygotes (Figure 3C). The genotype ratio of wild-type and heterozygotes was 1 to 1.94 (85/167) in the progenies of the self-pollinated plants, consistent with the expected ratio 1:2 from a single mutational event (*x*^2^ = 0.9248,*p* < 0.05) (Figure 3C), suggesting that the homozygous mutant *atprpl18* might be aborted during seed development. To address this hypothesis, siliques from wild-type and *atprpl18/+* plants were collected and analyzed at the indicated days after self-pollination. The appearance of the mutant silique was similar to that of wild-type plants. However, a large number of albino seeds (24.6%) were found randomly distributed in the siliques of *atprpl18/+* plants, whereas all seeds of the wild-type plants were green in color (Figure 3F). The albino seeds finally became shrunken in dark brown color and dried out and died in mature siliques. Reciprocal cross analysis of *atprpl18/+* plant showed that the male transmission efficiency was 84% (86/102) and the female transmission efficiency was 96% (88/92), both of them had no significant differences from the expected value 100% (Table 1), indicating that *AtPRPL18* did not affect gametophyte development. Together, these results suggested that the missing of the *atprpl18* homozygous offspring from *atprpl18/+* self-pollination was caused by impaired seed development.

**TABLE 1:** The gamete transmission efficiency of *atprpl18/+* mutant.

♀ × ♂	*atprpl18/+*	*+/+*	Ratio	TE	Expected	*P*-value
*atprpl18/+* × WT	88	92	1.045	0.96	1	0.8330
WT × *atprpl18/+*	86	102	1.186	0.84	1	0.4089

To further confirm that the seed abortion phenotype was caused by *AtPRPL18* mutation, CRISPR/Cas9 gene editing experiment was performed to generate *AtPRPL18* mutants. A series of heterozygous knockout mutants were obtained from the T1 transgenic plants (Figure 3D). The T2 plants of *ko1*/+(*knockout 1*) without T-DNA were identified for further study (Supplementary Figure 2). Consistent with the findings in *atprpl18/+* mutant, albino seeds were observed in the 9-day siliques of *ko1/+* plants, and homozygous mutant could not be detected from the offspring of *ko1/+* plants (Figures 3F,G). Additionally, a construct containing the genomic DNA fragment encompassing the *AtPRPL18* gene body was generated and transformed into *atprpl18/+* mutant. Heterozygous mutant plants complemented by the transgene, *atprpl18/+* COM, were identified by PCR and sequencing analysis. *atprpl18/+* COM plants exhibited a similar vegetative appearance to wild-type plants, and had only 5% (*n* = 1098) albino seeds compared the expected value 5.75% (Figures 3F,G). These results indicated that *AtPRPL18* is essential for seed development. However, plants overexpressing *AtPRPL18* did not show any significant difference from wild-type plants (Figure 3E and Supplementary Figure 3).

### Embryo development of *atprpl18/+* in Arabidopsis

**FIGURE 4:**
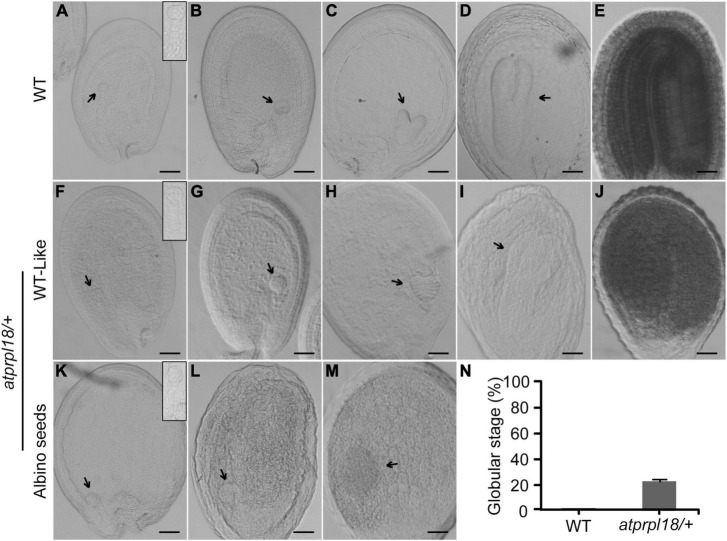
Comparison of the embryo development in WT and *atprpl18/+* mutant. **(A–E)** Various developmental stages of WT embryos. **(A)** 4-cell, **(B)** globular, **(C)** heart, **(D)** torpedo, **(E)** mature. **(F–J)** WT-like embryos from the developing siliques of *atprpl18/+* mutant. **(K–M)** Abnormal embryos from the albino seeds of *atprpl18/+* mutant. The magnified views of embryos from **(A,F,K)** were shown on the top right of the graphs. **(N)** The rate of arrested embryos in the 9 DAF siliques of WT and *atprpl18/+* mutant plants. Eight siliques for each genotype plants were examined. The rate of embryos arrested at globular stage per silique was calculated. Data represent means ± SD. This experiment was repeated twice with similar results. Scale bars = 50 μm **(A–M)**.

To investigate the function of *AtPRPL18* in seed development, successive stages of embryo development were examined in wild-type and the *atprpl18/+* mutant plants. In wild-type plants, the embryos underwent a series of development stages to become mature embryos (Figures 4A–E). There was no detectable difference in embryo development between WT and the *atprpl18/+* mutant in the first 2 days after fertilization (before heart stage) (Figures 4A,B,F,G). However, on the third day after fertilization(3 DAF), some seeds appeared in white color, and the others were in green color. The green seeds from wild-type and *atprpl18/+* mutant developed into late globular and early heart stages, while all of the embryos in albino seeds remained at globular stage (Figures 4C,H,K). When embryos in green seeds developed into torpedo stage, even finished embryogenesis (Figures 4D,E,I,J), the embryos in albino seeds were still at globular stage but with enlarged irregular structure (Figures 4L–M). On the 9th DAF, almost all of the embryos from wild-type plants were established, whereas, about 22.01% seeds of *atprpl18/+* mutant were arrested at globular stage (Figure 4N). These data revealed that *AtPRPL18* is indispensable for embryo development in the transition from globular stage to heart stage in Arabidopsis.

### *OsPRPL18* is required for chloroplast development in rice

**FIGURE 5:**
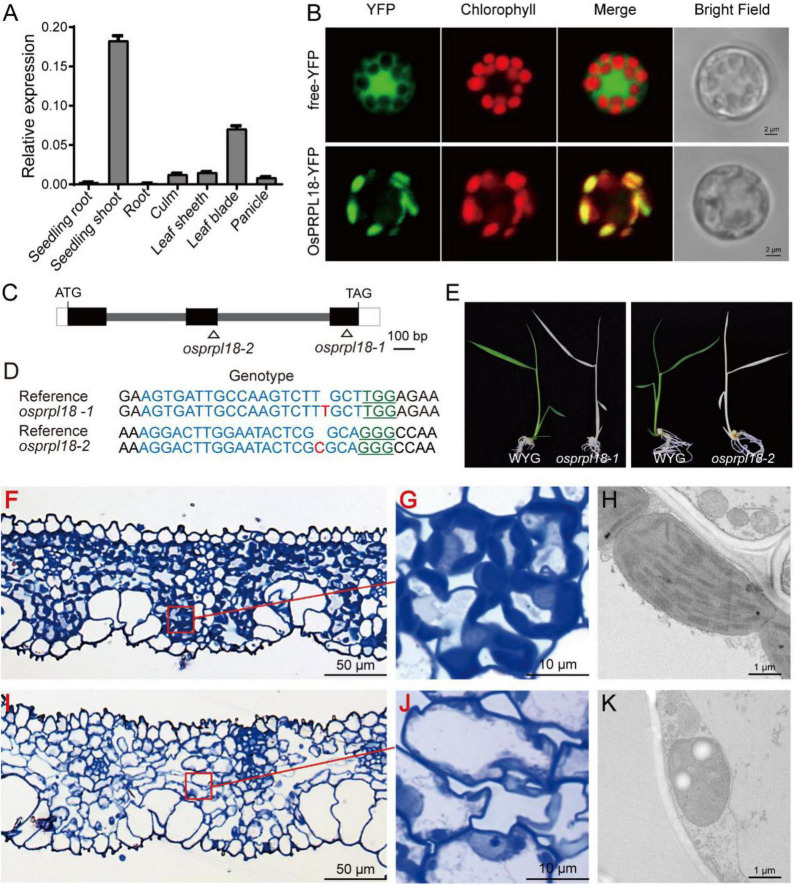
Characterization of the function of *OsPRPL18* in rice. **(A)** The gene expression of *OsPRPL18* in various rice tissues. UBQ served as the internal control. Data represent means ± SD from three biological replicates. **(B)** Subcellular location of OsPRPL18-YFP in rice protoplast. Free YFP was used as a control. The chlorophyll autofluorescence indicates chloroplast. Scale bars = 5 μm **(C)** The gene structure of *OsPRPL18.* **(D)** The genotypes of *osprpl18* mutants. The guide RNA targeted DNA sequence and PAM motif were highlighted in blue and green, respectively. The targeted site was marked as triangle in **(C)**. **(E)** 11-day-old seedlings of wild-type rice (WYG) and *osprpl18-1* and *osprpl18-2* mutants. **(F–K)** The structure of leaf blade and chloroplast of WYG **(F–G)** and the *osprpl18-1* mutant **(I–K)**.

Dicotyledonous plants and monocotyledonous plants are very distinct in embryo patterning and seed structure (Radoeva et al., 2019). To compare the biological functions of PRPL18 proteins in dicotyledonous plants and monocotyledonous plants, we further characterized the function of *OsPRPL18* in the model monocotyledonous plant rice. The real-time RT-PCR analysis revealed that *OsPRPL18* was expressed highly in seedling shoot and leaf sheath, lower in leaf blade, stem and panicle, and very low in root (Figure 5A), suggesting that *OsPRPL18* may be critical for green tissues. Protein subcellular localization analysis showed that the fusion proteins OsPRPL18-YFP and OsPRPL18-eGFP were colocalized with the autofluorescence of chlorophyll in rice protoplast and tobacco leaf cells, respectively, indicating that OsPRPL18 is a chloroplast-localized protein (Figure 5B and Supplementary Figure 1B).

To test the function of *OsPRPL18* in rice, CRISPR/Cas9-based gene editing constructs targeting the second or the third exon of *OsPRPL18* were introduced into wild-type rice (WYG) by Agrobacteria-mediated rice transformation. Two independent homozygous mutants were identified, in which the *OsPRPL18* gene exhibited frame shift mutation (Figures 5C,D). However, these homozygous mutants displayed albino seedling phenotypes and died at the three-leaf stage (Figure 5E).

The albino leaf phenotype was usually caused by an impairment of chloroplast (Puthur et al., 2013). To examine the chloroplast development in *osprpl18-1* plants, the albino leaves were cross-sectioned and analyzed using light microscope and TEM, respectively. In the semi-thin sections of wild-type leaves, chloroplasts were stained in dark blue by toluidine blue (Figures 5F,G), whereas no typical chloroplast-like structure was observed in *osprpl18-1* plants under light microscope (Figures 5I,J). Interestingly, chloroplast-like structures were clearly visible in the *osprpl18-1* mutant under TEM (Figure 5K). However, these chloroplast-like structures did not contain any thylakoids, compared with the well-developed chloroplasts with stacked grana and thylakoid membranes in wide-type plants (Figures 5H,K). These results suggested that *OsPRPL18* is required for chloroplast development in rice.

### Plastid ribosome biogenesis is impaired in the *osprpl18-1* mutant

**FIGURE 6:**
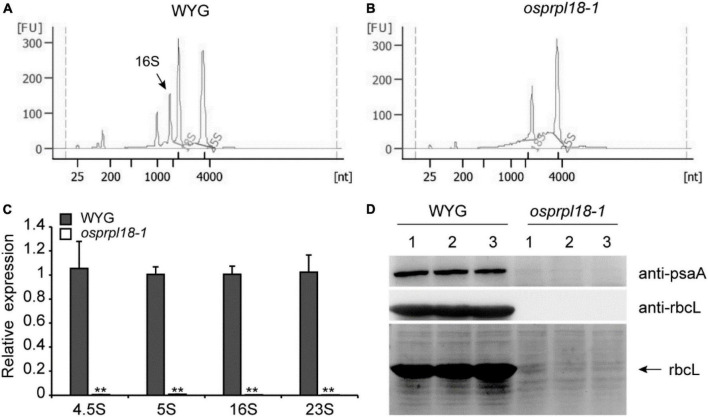
Comparison of the expression level of plastid rRNA and plastid-encoded protein rbcL in the seedling shoots of WYG and *osprpl18-1* mutant. rRNA analysis of WYG **(A)** and *osprpl18-1* mutant **(B)** with an Agilent 2,100 bioanalyzer. **(C)** qRT-PCR analysis of the transcripts level of plastid rRNA. UBQ served as the internal control. Gene expression values are presented relative to WYG levels (set as 1). Data represent means ± SD from three biological replicates (**P < 0.01; Student’s *t*-test). **(D)** Western blot analyses of the expression of rbcL and psaA. Immunoblotting was performed with specific antibodies, anti-rbcL and anti-psaA (Beijing Protein Innovation Co., Ltd, AbP80037-A-SE, AbP80033-A-SE), respectively (upper panels). The coomassie blue stained gel was used as a loading control (lower panel). For each genotype, three independent biological samples were analyzed.

Ribosomal proteins are essential for maintaining rRNA stability and ribosome assembly (Saez-Vasquez and Delseny, 2019). The ribosomal protein L18 directly interacted with 5S rRNA and 23S rRNA, thus incorporating the 5S rRNA into the 50S ribosomal subunit (Newberry and Garrett, 1980). There are four kinds of rRNA in plastid, including 4.5S, 5S, 16S, and 23S rRNA (Stern et al., 2010). To assess the effects of OsPRPL18 in plastid ribosome biogenesis, rRNA was examined by an Agilent 2,100 bioanalyzer. It was clearly to identify the peak of cytosolic 18S rRNA and 25S rRNA, and the peak of plastid 16S rRNA in the total RNA sample of wild-type (WYG) (Figure 6A), whereas the amount of plastid 16S rRNA was barely detectable in the *osprpl18-1* mutant (Figure 6B). qRT-PCR analysis showed that the transcripts of plastid rRNAs were dramatically reduced in the *osprpl18-1* mutant (Figure 6C). rbcL (large subunit of the ribulose-bisphosphate carboxylase), the most abundant plant protein, is encoded by a plastid gene and synthesized by chloroplast translation apparatus (Vitlin Gruber and Feiz, 2018). The plastid-encoded protein psaA comprises the reaction center for photosystem I along with psaB (Kapoor et al., 1994). Western blotting analyses revealed that the synthesis of rbcL and psaA were almost aborted in the *osprpl18-1* mutant (Figure 6D). Collectively, these data suggested that OsPRPL18 is indispensable for plastid ribosome biogenesis.

### The *osprpl18-1* mutant is defective in the intron splicing in plastid

**FIGURE 7:**
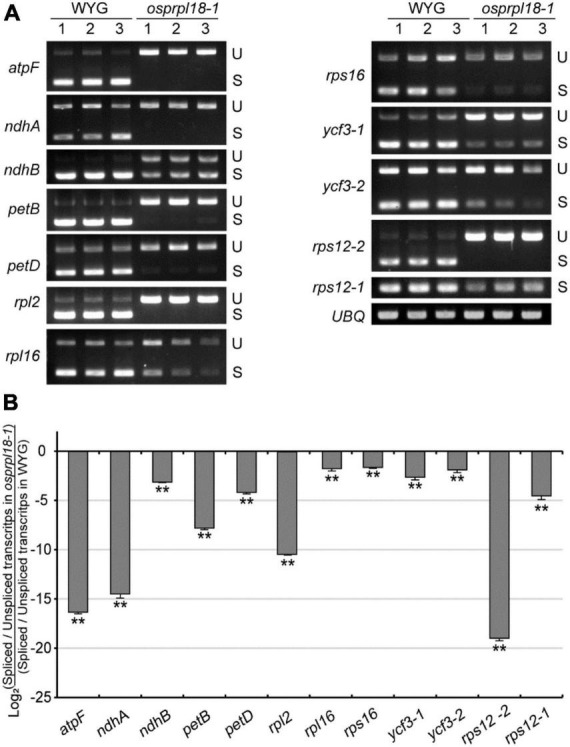
Comparison of the efficiency of intron splicing in the plastids of WYG and *osprpl18-1* mutant. **(A)** Spliced (S) and unspliced (U) transcripts examined by RT-PCR. **(B)** Relative quantitative analyses of plastid intron splicing efficiency of WYG and *osprpl18-1* mutant by qRT-PCR. Data represent the mean log2 ratios of splicing efficiencies in *osprpl18-1* mutant to the wild type (WYG). Three independent biological replicates were used for each genotype. The asterisks indicate significant difference between WYG and *osprpl18-1* mutant (**P < 0.01; Student’s *t*-test).

Plant plastid genome harbors two main types of introns, group I and group II, defined by their different splicing mechanisms and conserved structural elements (Stern et al., 2010). In land plants, there are 18 introns distributed in 10 protein-encoding genes and 6 tRNA genes in plastid genome (Hess et al., 1994), and only *rps12* contains a trans-splicing intron (Koller et al., 1987). Recent study revealed that two homologs of AtPRPL18, μL18-L1 and μL18-L8, were necessary for the splicing of certain mitochondrial and plastid group II introns, respectively (Wang et al., 2020). μL18-L8 was required for the trans splicing of the first intron of *rps12* in Arabidopsis plastid. To test whether OsPRPL18 is involved in the intron splicing in plastid, intron splicing efficiency of the representative genes were examined by RT-PCR and qRT-PCR. Surprisingly, the trans-splicing efficiency of the first intron of *rps12* was also remarkably reduced in the *osprpl18-1* mutant (Figures 7A,B). Furthermore, all of the other tested genes also showed obvious reductions of intron splicing efficiency in the *osprpl18-1* mutant compared with that in wild-type (WYG) (Figure 7). These results indicated that OsPRPL18 is required for intron splicing in rice plastid.

## Discussion

Plastid is a semiautonomous organelle. Lots of essential plastid proteins are translated by plastid ribosomes. PRPs are fundamental components of plastid ribosome, which play diverse roles in plant viability and development (Tiller and Bock, 2014). In this study, we report the different functions of PRPL18s in Arabidopsis and rice. Both of AtPRPL18 and OsPRPL18 were essential for plant development. However, knockout of *AtPRPL18* retarded the transition from globular stage to heart stage in Arabidopsis, while knockout of *OsPRPL18* did not affect embryo development, but resulted in an albino lethal phenotype at seedling stage in rice.

### PRPL18s are essential for plant viability

In bacteria, ribosome is consisted of a 50S large subunit and a 30S small subunit. The 50S subunit contains 23S rRNA, 5S rRNA and 34 ribosomal proteins. The 5S rRNA collaborates with ribosomal large subunit protein L5, L18 and L25 to form an essential autonomous structural domain of 50S subunit. L5 and L18 were very important for the incorporation of 5S rRNA into 50S ribosomal subunit (Newberry and Garrett, 1980). The C-terminal part of L18 functions in binding with 5S rRNA, and its N-terminal region is involved in the interaction of 5S rRNA with 23S rRNA (Tiller and Bock, 2014). Disruption of the coding genes of L5, L18 and L25 revealed that L5 and L18 but not L25 were essential for *E. coli* viability (Korepanov et al., 2007). Plants have two semiautonomous organelles, chloroplast and mitochondria, which are proposed to be evolved from their prokaryotic ancestors. The structure and constitution of ribosomes of chloroplast and mitochondria are similar to that in bacteria (Zoschke and Bock, 2018). Protein sequence-based search for L18 homologues in Arabidopsis found two close homologs, AtPRPL18/μL18c and μL18m (Wang et al., 2020). μL18m was localized in mitochondria and appeared to be a mitochondrial ribosomal protein. AtPRPL18 was localized in chloroplast and was proposed to be a plastid ribosomal protein (Wang et al., 2020). Phylogenetic analysis showed that the plastid ribosomal protein L18s were very conserved in plant kingdom (Figure 1), indicating they may have a similar molecular function. Gene expression analyses showed both of *OsPRPL18* and *AtPRPL18* were highly expressed in green tissues, and their encoding proteins were localized in chloroplast. In the heterozygous mutants *atprpl18/+* and *ko-1/+* of *AtPRPL18*, about a quarter of albino developing seeds were observed in silique (Figures 3F,G). Cytological analysis revealed the embryos were arrested at globular stage in these albino seeds (Figure 4). The albino seeds were then shrunken and aborted. Genetic and complementation analyses showed the albino seed phenotype was caused by the mutation of *AtPRPL18*, suggesting AtPRPL18 was essential for Arabidopsis seed development. Moreover, knockout of *OsPRPL18* led to an albino lethality of rice seedlings (Figure 5E). Cytological analysis found that the chloroplast development was impaired in the leaves of *osprpl18-1* mutants (Figure 5K). Further studies uncovered the indispensable role of OsPRPL18 in plastid ribosome biogenesis (Figure 6). Overall, consistent with the function of L18 in *E. coli*, PRPL18s are essential for plant viability.

### Loss-function of PRPL18s lead to different consequences during embryogenesis in Arabidopsis and rice

Because of the conserved evolutionary relationship of the ribosomes in bacteria and plastid, lots of bacterial ribosomal proteins and its plant homologs were proved to be essential for bacteria and plant viability respectively, such as S2, S3, S4, S5, S9, S13, S14, S16, S18, L3, L4, L6, L10, L13, L18, L20, L22, L23, and L35 and their plant homologs (Ahlert et al., 2003; Rogalski et al., 2006; Baba et al., 2008; Bryant et al., 2011; Fleischmann et al., 2011; Shoji et al., 2011; Romani et al., 2012). However, some other ribosomal proteins are in a different case. For example, S9, S13, S20, L1, L21, L27, L28, and L31 were shown to be dispensable in bacteria, but their homologs were essential for plant viability (Dabbs, 1991; Bubunenko et al., 2007; Baba et al., 2008; Bryant et al., 2011; Shoji et al., 2011; Romani et al., 2012; Yin et al., 2012). On the contrary, S1 was proved to be essential in bacteria, but its homolog was reported to be non-essential for plant viability (Baba et al., 2008; Bryant et al., 2011; Romani et al., 2012). Interestingly, even in plants, some PRPs and their homologs also appeared to have diverse functions. In the siliques of the Arabidopsis heterozygous mutant *atprps20/+*, the albino/aborted seeds accounted for about one quarter of the total seeds, of which the embryos were arrested at globular stage (Romani et al., 2012). However, the null mutant of *OsPRPS20* was defective in chloroplast development and exhibited an albino lethal phenotype at seedling stage (Gong et al., 2013). Similar to the case of PRPS20, albino/aborted seeds were also observed in the *atprps9/+*, *atprpl13/+*, and *atprpl21/+* plants in Arabidopsis (Bryant et al., 2011; Yin et al., 2012), while an albino lethal phenotype was observed in the seedlings of null mutants *osprps9*, *osprpl13* and *osprpl21* in rice (Song et al., 2014; Lin et al., 2015; Qiu et al., 2018). In this study, we revealed that knockouts of *PRPL18s* in Arabidopsis and rice resulted in different phenotypical consequences. AtPRPL18 was essential for the transition of global stage to heart stage during embryo development, while OsPRPL18 did not affect embryogenesis. However, the *osprp18* mutant was impaired in chloroplast developing, and displayed an albino lethal phenotype at seedling stage. In Arabidopsis, plastids are few in zygote, but gradually increase in number during embryo development. At the globular stage, plastids differentiate into chloroplasts with chlorophyll accumulation, and thereby embryos start greening (Tejos et al., 2010). It was proposed that chloroplasts are vital in supplying nutrition to the developing embryo, therefore deficiency of chloroplast may disrupt embryogenesis in Arabidopsis (Tiller and Bock, 2014). In contrast, embryo development is largely dependent on the nutrients supplied from endosperm in rice (An et al., 2020). Thus, Arabidopsis may be more susceptible to the loss of PRPL18 than rice during embryogenesis. Intriguingly, although OsPRPS9 and its maize homolog ZmPRPS9 share 82% identity, OsPRPS9 was proved to be a plastid protein, while ZmPRPS9 was shown to be localized on both chloroplast and nucleus (Ma and Dooner, 2004; Qiu et al., 2018). Moreover, OsPRPS9 was dispensable for rice embryogenesis, while the *zmprps9* mutant exhibited an early embryo lethal phenotype (Ma and Dooner, 2004; Qiu et al., 2018). So, another possibility is that PRPL18 might have evolved species-specific functions. To address these hypothesis, future works on the molecular functions of PRPs and heterological complementary works are required.

### PRPL18s may be involved in intron splicing in plastid

The intron splicing efficiency in plastid is largely dependent on the plastid-encoded components. The intron splicing of *rps2* and *rps12* were impaired in the barley mutants with ribosome-deficient plastids (Hess et al., 1994; Hubschmann et al., 1996). In land plants, the intron in the *trnK* gene encodes a conserved maturase called MatK (Maturase K) (Zoschke et al., 2010). MatK directly interacts with the introns of *trnA*, *trnI*, *trnV*, *trnK*, *atpF*, *rpl2* and the second intron of *rps12* (Zoschke et al., 2010). Application of heterologously expressed MatK protein increased the intron self-splicing efficiency of the second intron of *rps12 in vitro* (Barthet et al., 2020). Thus, MatK was proposed to be a plastid-encoded splicing factor. However, addition of MatK protein did not increased the intron self-splicing efficiency of *rpl2* (Barthet et al., 2020), and a functioning translational apparatus was not prerequisite for the intron splicing of *rps16*, *rpl16*, *ndhB*, *petD*, and *trnL* (Hess et al., 1994), suggesting the presence of other factors in the regulation of plastid intron splicing. Ribosomal proteins maybe participate in intron splicing. Human ribosomal protein S13 was reported to inhibit excision of *RPS13* intron 1 *in vitro* (Malygin et al., 2007). Ribosomal protein L10a regulated its own alternative pre-mRNA splicing by directly and specifically binds to an evolutionarily conserved stretch between the two alternative 5’ splice sites in pre-mRNA to switch the splice site choice (Takei et al., 2016). A recent study reported that two homologs of PRPL18, μL18-L1, and μL18-L8, play essential roles in the splicing of the fourth intron of *nad5* pre-mRNAs in mitochondria, and the removal of *rps12* intron 1 in Arabidopsis plastid, respectively (Wang et al., 2020). μL18-L1 and μL18-L8 specifically associate with *nad5* intron 4 and *rps12* intron 1, respectively, indicating direct roles in the splicing of their targeted introns (Wang et al., 2020). Nevertheless, the function of PRPL18s in intron splicing remains elusive. In this report, we found that loss-function of OsPRPL18 resulted in a serious reduction of the intron splicing efficiency of all of the tested plastid introns, As the biogenesis of chloroplast ribosome was defective in *osprpl18-1*, OsPRPL18 may affect intron splicing by maintaining biosynthesis of the regulatory proteins such as MatK and so on. Alternatively, OsPRPL18 may participate in the process of plastid intron splicing directly. Employing the *in vitro* activity assay to test the role of OsPRPL18 in plastid intron excision would be helpful to uncover the direct function of OsPRPL18 in plastid intron splicing (Barthet et al., 2020).

In summary, our results provide the evidence that PRPL18s are essential for plant viability. However, AtPRPL18 is required for embryogenesis in Arabidopsis, while OsPRPL18 is dispensable for embryogenesis but required for seedling development in rice, although the underlying mechanisms need to be further clarified. Moreover, the surprising finding of the prerequisite role of OsPRPL18 in plastid intron splicing highlights the functions of ribosomal proteins in other biological process.

## Data availability statement

The datasets presented in this study can be found in online repositories. The names of the repository/repositories and accession number(s) can be found in the article/Supplementary material.

## Author contributions

JW, XT, and SC designed the experiments and wrote the manuscript. SC, XZ, JW, YQL, SQ, XP, XX, YJL, and CL performed the experiments. SC and JW analyzed the data. All authors read and approved the manuscript for publication.

## Footnotes

1
https://www.ncbi.nlm.nih.gov/

2
https://phytozome-next.jgi.doe.gov/

3
https://www.ebi.ac.uk/Tools/msa/clustalo/

4
http://www.jalview.org/

5
http://abrc.osu.edu

## Supplementary material

Supplementary Figure 1 Subcellular localizations of AtPRPL18-GFP and OsPRPL18-GFP in *N. benthamiana* leaf cells.

Supplementary Figure 2 Identification of the T-DNA free *ko-1*/+ mutant plants.

Supplementary Figure 3 Analyses of the transcript levels of *AtPRPL18* in OE-1 and OE-2.

## References

[B1] Ahlert D. Ruf S. Bock R. (2003). Plastid protein synthesis is required for plant development in tobacco. *Proc. Natl. Acad. Sci. U.S.A.* 100 15730–15735. 10.1073/pnas.2533668100 14660796 PMC307636

[B2] Almagro Armenteros J. J. Salvatore M. Emanuelsson O. Winther O. Von Heijne G. Elofsson A. (2019). Detecting sequence signals in targeting peptides using deep learning. *Life Sci. Alliance* 2:e201900429. 10.26508/lsa.201900429 31570514 PMC6769257

[B3] An L. Tao Y. Chen H. He M. Xiao F. Li G. (2020). Embryo-Endosperm interaction and its agronomic relevance to rice quality. *Front. Plant Sci.* 11:587641. 10.3389/fpls.2020.587641 33424883 PMC7793959

[B4] Baba T. Huan H. C. Datsenko K. Wanner B. L. Mori H. (2008). The applications of systematic in-frame, single-gene knockout mutant collection of *Escherichia coli* K-12. *Methods Mol. Biol.* 416 183–194. 10.1007/978-1-59745-321-9_12 18392968

[B5] Barthet M. M. Pierpont C. L. Tavernier E. K. (2020). Unraveling the role of the enigmatic MatK maturase in chloroplast group IIA intron excision. *Plant Direct* 4:e00208. 10.1002/pld3.208 32185246 PMC7068846

[B6] Brosius J. Schiltz E. Chen R. (1975). The primary structure of the 5S RNA binding protein L18 from *Escherichia coli* ribosomes. *FEBS Lett.* 56 359–361. 10.1016/0014-5793(75)81127-6 1098937

[B7] Bryant N. Lloyd J. Sweeney C. Myouga F. Meinke D. (2011). Identification of nuclear genes encoding chloroplast-localized proteins required for embryo development in *Arabidopsis*. *Plant Physiol.* 155 1678–1689. 10.1104/pp.110.168120 21139083 PMC3091104

[B8] Bubunenko M. Baker T. Court D. L. (2007). Essentiality of ribosomal and transcription antitermination proteins analyzed by systematic gene replacement in *Escherichia coli*. *J. Bacteriol.* 189 2844–2853. 10.1128/JB.01713-06 17277072 PMC1855809

[B9] Chang Z. Chen Z. Wang N. Xie G. Lu J. Yan W. (2016). Construction of a male sterility system for hybrid rice breeding and seed production using a nuclear male sterility gene. *Proc. Natl. Acad. Sci. U.S.A.* 113 14145–14150. 10.1073/pnas.1613792113 27864513 PMC5150371

[B10] Chen H. Zou W. Zhao J. (2015). Ribonuclease J is required for chloroplast and embryo development in *Arabidopsis*. *J. Exp. Bot.* 66 2079–2091. 10.1093/jxb/erv010 25871650 PMC4378637

[B11] Chen S. Tao L. Zeng L. Vega-Sanchez M. E. Umemura K. Wang G. L. (2006). A highly efficient transient protoplast system for analyzing defence gene expression and protein-protein interactions in rice. *Mol. Plant Pathol.* 7 417–427. 10.1111/j.1364-3703.2006.00346.x 20507457

[B12] Dabbs E. R. (1991). Mutants lacking individual ribosomal proteins as a tool to investigate ribosomal properties. *Biochimie* 73 639–645. 10.1016/0300-9084(91)90043-z 1837238

[B13] Fleischmann T. T. Scharff L. B. Alkatib S. Hasdorf S. Schottler M. A. Bock R. (2011). Nonessential plastid-encoded ribosomal proteins in tobacco: a developmental role for plastid translation and implications for reductive genome evolution. *Plant Cell* 23 3137–3155. 10.1105/tpc.111.088906 21934145 PMC3203423

[B14] Gong X. Jiang Q. Xu J. Zhang J. Teng S. Lin D. (2013). Disruption of the rice plastid ribosomal protein s20 leads to chloroplast developmental defects and seedling lethality. *G3* 3 1769–1777. 10.1534/g3.113.007856 23979931 PMC3789801

[B15] Hess W. R. Hoch B. Zeltz P. Hubschmann T. Kossel H. Borner T. (1994). Inefficient rpl2 splicing in barley mutants with ribosome-deficient plastids. *Plant Cell* 6 1455–1465. 10.1105/tpc.6.10.1455 7994178 PMC160533

[B16] Hubschmann T. Hess W. R. Borner T. (1996). Impaired splicing of the rps12 transcript in ribosome-deficient plastids. *Plant Mol. Biol.* 30 109–123. 10.1007/BF00017806 8616228

[B17] Jefferson R. A. Kavanagh T. A. Bevan M. W. (1987). GUS fusions: beta-glucuronidase as a sensitive and versatile gene fusion marker in higher plants. *EMBO J.* 6 3901–3907. 10.1002/j.1460-2075.1987.tb02730.x 3327686 PMC553867

[B18] Jensen P. E. Leister D. (2014). Chloroplast evolution, structure and functions. *F1000Prime Rep.* 6:40. 10.12703/P6-40 24991417 PMC4075315

[B19] Kapoor S. Maheshwari S. C. Tyagi A. K. (1994). Developmental and light-dependent cues interact to establish steady-state levels of transcripts for photosynthesis-related genes (psbA, psbD, psaA and rbcL) in rice (*Oryza sativa L*.). *Curr. Genet.* 25 362–366. 10.1007/BF00351491 8082180

[B20] Koller B. Fromm H. Galun E. Edelman M. (1987). Evidence for in vivo trans splicing of pre-mRNAs in tobacco chloroplasts. *Cell* 48 111–119. 10.1016/0092-8674(87)90361-8 3791410

[B21] Korepanov A. P. Gongadze G. M. Garber M. B. Court D. L. Bubunenko M. G. (2007). Importance of the 5 S rRNA-binding ribosomal proteins for cell viability and translation in *Escherichia coli*. *J. Mol. Biol.* 366 1199–1208. 10.1016/j.jmb.2006.11.097 17198710 PMC1939977

[B22] Lin D. Jiang Q. Zheng K. Chen S. Zhou H. Gong X. (2015). Mutation of the rice ASL2 gene encoding plastid ribosomal protein L21 causes chloroplast developmental defects and seedling death. *Plant Biol.* 17 599–607. 10.1111/plb.12271 25280352

[B23] Lv J. Shang L. Chen Y. Han Y. Yang X. Xie S. (2020). OsSLC1 encodes a pentatricopeptide repeat protein essential for early chloroplast development and seedling survival. *Rice* 13:25. 10.1186/s12284-020-00385-5 32297039 PMC7160225

[B24] Ma X. Zhang Q. Zhu Q. Liu W. Chen Y. Qiu R. (2015). A robust CRISPR/Cas9 system for convenient, high-efficiency multiplex genome editing in monocot and dicot plants. *Mol. Plant* 8 1274–1284. 10.1016/j.molp.2015.04.007 25917172

[B25] Ma Z. Dooner H. K. (2004). A mutation in the nuclear-encoded plastid ribosomal protein S9 leads to early embryo lethality in maize. *Plant J.* 37 92–103. 10.1046/j.1365-313x.2003.01942.x 14675435

[B26] Malygin A. A. Parakhnevitch N. M. Ivanov A. V. Eperon I. C. Karpova G. G. (2007). Human ribosomal protein S13 regulates expression of its own gene at the splicing step by a feedback mechanism. *Nucleic Acids Res.* 35 6414–6423. 10.1093/nar/gkm701 17881366 PMC2095825

[B27] Morita-Yamamuro C. Tsutsui T. Tanaka A. Yamaguchi J. (2004). Knock-out of the plastid ribosomal protein S21 causes impaired photosynthesis and sugar-response during germination and seedling development in *Arabidopsis thaliana*. *Plant Cell Physiol.* 45 781–788. 10.1093/pcp/pch093 15215513

[B28] Newberry V. Garrett R. A. (1980). The role of the basic N-terminal region of protein L18 in 5S RNA-23S RNA complex formation. *Nucleic Acids Res.* 8 4131–4142. 10.1093/nar/8.18.4131 6159586 PMC324224

[B29] Pesaresi P. Varotto C. Meurer J. Jahns P. Salamini F. Leister D. (2001). Knock-out of the plastid ribosomal protein L11 in Arabidopsis: effects on mRNA translation and photosynthesis. *Plant J.* 27 179–189. 10.1046/j.1365-313x.2001.01076.x 11532164

[B30] Puthur J. T. Shackira A. M. Saradhi P. P. Bartels D. (2013). Chloroembryos: a unique photosynthesis system. *J. Plant Physiol.* 170 1131–1138. 10.1016/j.jplph.2013.04.011 23706538

[B31] Qiu Z. Chen D. He L. Zhang S. Yang Z. Zhang Y. (2018). The rice white green leaf 2 gene causes defects in chloroplast development and affects the plastid ribosomal protein S9. *Rice* 11:39. 10.1186/s12284-018-0233-2 29995230 PMC6041223

[B32] Radoeva T. Vaddepalli P. Zhang Z. Weijers D. (2019). Evolution, initiation, and diversity in early plant embryogenesis. *Dev. Cell* 50 533–543. 10.1016/j.devcel.2019.07.011 31505175

[B33] Rogalski M. Ruf S. Bock R. (2006). Tobacco plastid ribosomal protein S18 is essential for cell survival. *Nucleic Acids Res.* 34 4537–4545. 10.1093/nar/gkl634 16945948 PMC1636375

[B34] Romani I. Tadini L. Rossi F. Masiero S. Pribil M. Jahns P. (2012). Versatile roles of *Arabidopsis* plastid ribosomal proteins in plant growth and development. *Plant J.* 72 922–934. 10.1111/tpj.12000 22900828

[B35] Saez-Vasquez J. Delseny M. (2019). Ribosome biogenesis in plants: from functional 45S ribosomal DNA organization to ribosome assembly factors. *Plant Cell* 31 1945–1967. 10.1105/tpc.18.00874 31239391 PMC6751116

[B36] Schmittgen T. D. Livak K. J. (2008). Analyzing real-time PCR data by the comparative C(T) method. *Nat. Protoc.* 3 1101–1108. 10.1038/nprot.2008.73 18546601

[B37] Shoji S. Dambacher C. M. Shajani Z. Williamson J. R. Schultz P. G. (2011). Systematic chromosomal deletion of bacterial ribosomal protein genes. *J. Mol. Biol.* 413 751–761. 10.1016/j.jmb.2011.09.004 21945294 PMC3694390

[B38] Song J. Wei X. Shao G. Sheng Z. Chen D. Liu C. (2014). The rice nuclear gene WLP1 encoding a chloroplast ribosome L13 protein is needed for chloroplast development in rice grown under low temperature conditions. *Plant Mol. Biol.* 84 301–314. 10.1007/s11103-013-0134-0 24132771

[B39] Sparkes I. A. Runions J. Kearns A. Hawes C. (2006). Rapid, transient expression of fluorescent fusion proteins in tobacco plants and generation of stably transformed plants. *Nat. Protoc.* 1 2019–2025. 10.1038/nprot.2006.286 17487191

[B40] Stern D. B. Goldschmidt-Clermont M. Hanson M. R. (2010). Chloroplast RNA metabolism. *Annu. Rev. Plant Biol.* 61 125–155. 10.1146/annurev-arplant-042809-112242 20192740

[B41] Takei S. Togo-Ohno M. Suzuki Y. Kuroyanagi H. (2016). Evolutionarily conserved autoregulation of alternative pre-mRNA splicing by ribosomal protein L10a. *Nucleic Acids Res.* 44 5585–5596. 10.1093/nar/gkw152 26961311 PMC4937301

[B42] Tamura K. Stecher G. Kumar S. (2021). MEGA11: molecular evolutionary genetics analysis version 11. *Mol. Biol. Evol.* 38 3022–3027. 10.1093/molbev/msab120 33892491 PMC8233496

[B43] Tejos R. I. Mercado A. V. Meisel L. A. (2010). Analysis of chlorophyll fluorescence reveals stage specific patterns of chloroplast-containing cells during *Arabidopsis* embryogenesis. *Biol. Res.* 43 99–111. 21157637

[B44] Tiller N. Bock R. (2014). The translational apparatus of plastids and its role in plant development. *Mol. Plant* 7 1105–1120. 10.1093/mp/ssu022 24589494 PMC4086613

[B45] Tiller N. Weingartner M. Thiele W. Maximova E. Schöttler M. A. Bock R. (2012). The plastid-specific ribosomal proteins of *Arabidopsis thaliana* can be divided into non-essential proteins and genuine ribosomal proteins. *Plant J.* 69 302–316. 10.1111/j.1365-313X.2011.04791.x 21923745

[B46] Toki S. Hara N. Ono K. Onodera H. Tagiri A. Oka S. (2006). Early infection of scutellum tissue with Agrobacterium allows high-speed transformation of rice. *Plant J.* 47 969–976. 10.1111/j.1365-313X.2006.02836.x 16961734

[B47] Tzafrir I. Pena-Muralla R. Dickerman A. Berg M. Rogers R. Hutchens S. (2004). Identification of genes required for embryo development in *Arabidopsis*. *Plant Physiol.* 135 1206–1220. 10.1104/pp.104.045179 15266054 PMC519041

[B48] Tzfira T. Tian G.-W. Lacroix B. T. Vyas S. Li J. Leitner-Dagan Y. (2005). PSAT vectors: a modular series of plasmids for autofluorescent protein tagging and expression of multiple genes in plants. *Plant Mol. Biol.* 57:503. 10.1007/s11103-005-0340-5 15821977

[B49] Vitlin Gruber A. Feiz L. (2018). Rubisco assembly in the chloroplast. *Front. Mol. Biosci.* 5:24. 10.3389/fmolb.2018.00024 29594130 PMC5859369

[B50] Waltz F. Nguyen T. T. Arrive M. Bochler A. Chicher J. Hammann P. (2019). Small is big in Arabidopsis mitochondrial ribosome. *Nat. Plants* 5 106–117. 10.1038/s41477-018-0339-y 30626926

[B51] Wang C. Fourdin R. Quadrado M. Dargel-Graffin C. Tolleter D. Macherel D. (2020). Rerouting of ribosomal proteins into splicing in plant organelles. *Proc. Natl. Acad. Sci. U.S.A.* 117 29979–29987. 10.1073/pnas.2004075117 33168708 PMC7703591

[B52] Wu J. X. Wu J. L. Yin J. Zheng P. Yao N. (2015). Ethylene modulates sphingolipid synthesis in *Arabidopsis*. *Front. Plant Sci.* 6:1122. 10.3389/fpls.2015.01122 26734030 PMC4679861

[B53] Yan L. Wei S. Wu Y. Hu R. Li H. Yang W. (2015). High-efficiency genome editing in *Arabidopsis* using YAO Promoter-driven CRISPR/Cas9 system. *Mol. Plant* 8 1820–1823. 10.1016/j.molp.2015.10.004 26524930

[B54] Yin T. Pan G. Liu H. Wu J. Li Y. Zhao Z. (2012). The chloroplast ribosomal protein L21 gene is essential for plastid development and embryogenesis in *Arabidopsis*. *Planta* 235 907–921. 10.1007/s00425-011-1547-0 22105802

[B55] Yoo S. D. Cho Y. H. Sheen J. (2007). *Arabidopsis* mesophyll protoplasts: a versatile cell system for transient gene expression analysis. *Nat. Protoc.* 2 1565–1572. 10.1038/nprot.2007.199 17585298

[B56] Zhang J. Yuan H. Yang Y. Fish T. Lyi S. M. Thannhauser T. W. (2016). Plastid ribosomal protein S5 is involved in photosynthesis, plant development, and cold stress tolerance in *Arabidopsis*. *J. Exp. Bot.* 67 2731–2744. 10.1093/jxb/erw106 27006483 PMC4861020

[B57] Zhang X. R. Henriques R. Lin S. S. Niu Q. W. Chua N. H. (2006). *Agrobacterium*-mediated transformation of *Arabidopsis thaliana* using the floral dip method. *Nat. Protoc.* 1 641–646. 10.1038/nprot.2006.97 17406292

[B58] Zhao D. S. Zhang C. Q. Li Q. F. Yang Q. Q. Gu M. H. Liu Q. Q. (2016). A residue substitution in the plastid ribosomal protein L12/AL1 produces defective plastid ribosome and causes early seedling lethality in rice. *Plant Mol. Biol.* 91 161–177. 10.1007/s11103-016-0453-z 26873698

[B59] Zhou K. Zhang C. Xia J. Yun P. Wang Y. Ma T. (2021). Albino seedling lethality 4; Chloroplast 30S ribosomal protein S1 is required for chloroplast ribosome biogenesis and early chloroplast development in rice. *Rice* 14:47. 10.1186/s12284-021-00491-y 34046768 PMC8160077

[B60] Zoschke R. Bock R. (2018). Chloroplast translation: structural and functional organization, operational control, and regulation. *Plant Cell* 30 745–770. 10.1105/tpc.18.00016 29610211 PMC5969280

[B61] Zoschke R. Nakamura M. Liere K. Sugiura M. Borner T. Schmitz-Linneweber C. (2010). An organellar maturase associates with multiple group II introns. *Proc. Natl. Acad. Sci. U.S.A.* 107 3245–3250. 10.1073/pnas.0909400107 20133623 PMC2840290

