# Supplementary figures and images for: The nuclear-encoded plastid ribosomal protein L18s are essential for plant development
Source: Front Plant Sci. 2022 Sep 23;13:949897. doi: 10.3389/fpls.2022.949897 (PMC9538462; doi:10.3389/fpls.2022.949897)

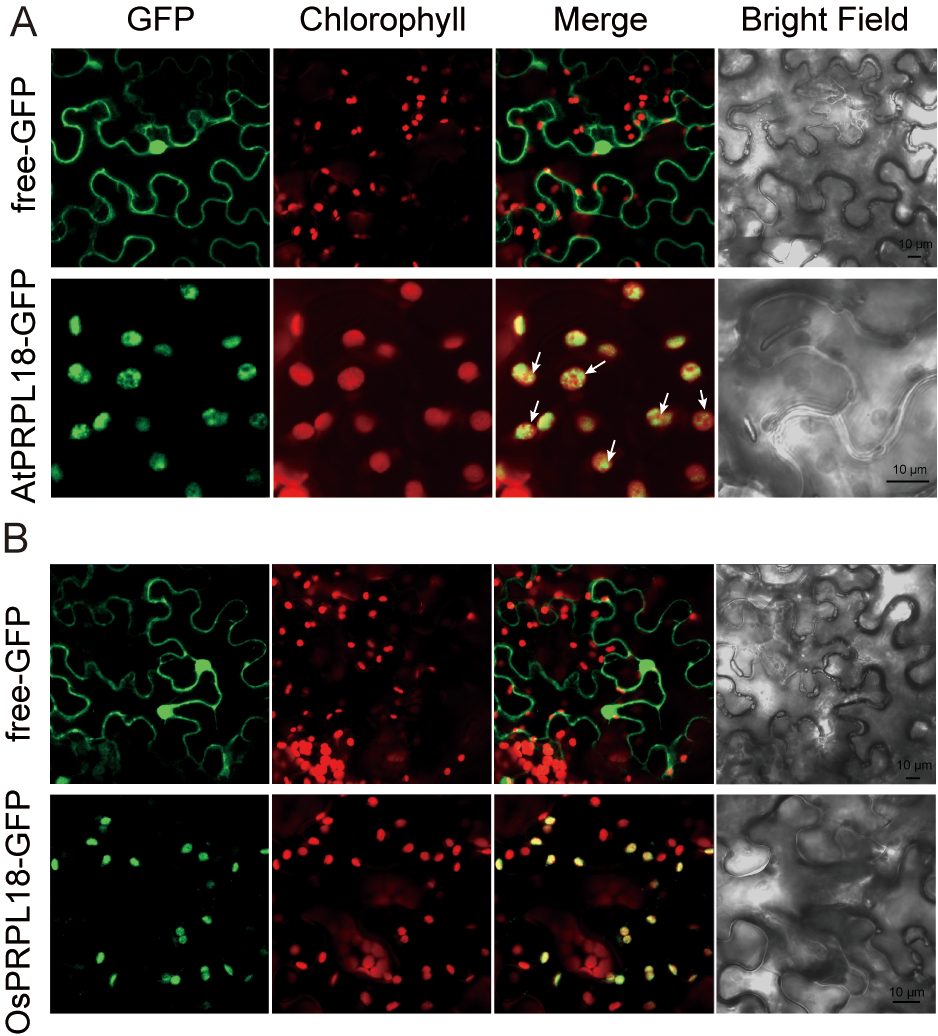

Supplement: Supplementary Figure 1 — Subcellular localizations of AtPRPL18-GFP and OsPRPL18-GFP in N. benthamiana leaf cells. [file Image_1.TIF]

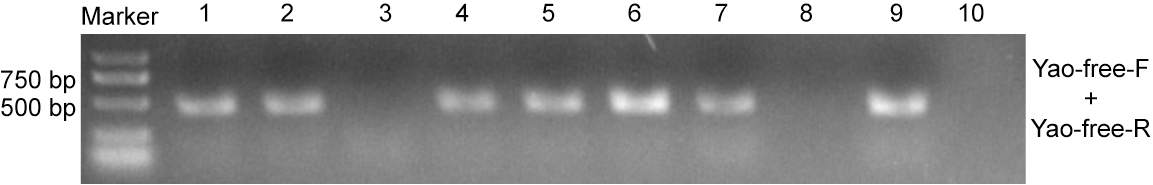

Supplement: Supplementary Figure 2 — Identification of the T-DNA free ko-1/+ mutant plants. [file Image_2.TIF]

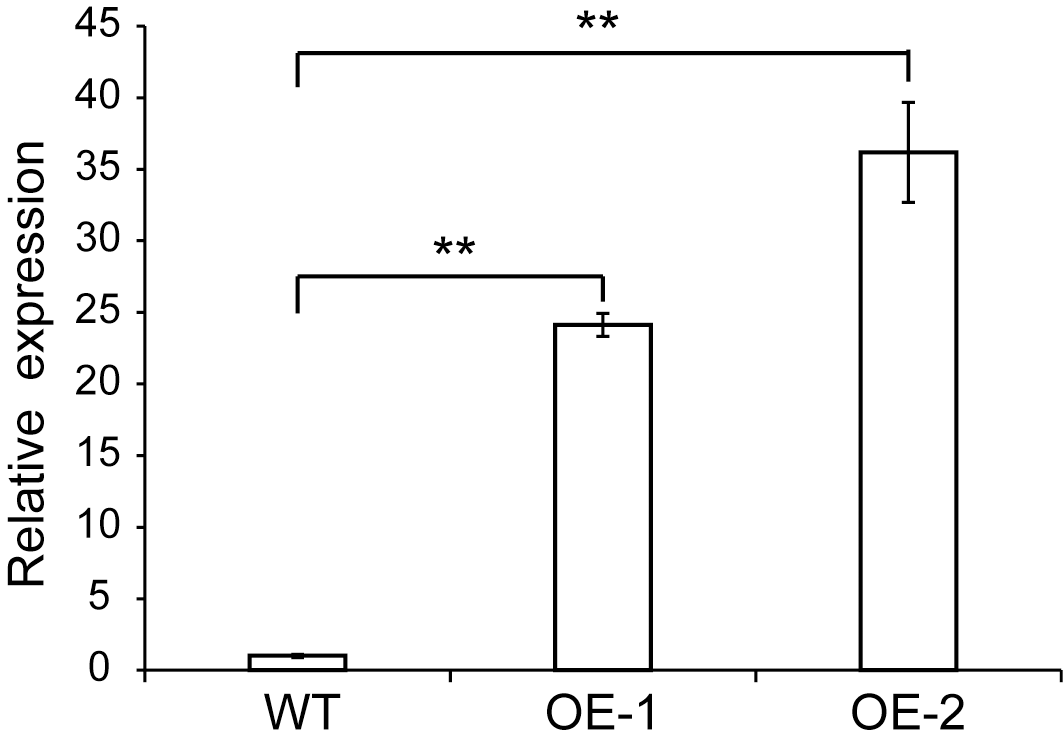

Supplement: Supplementary Figure 3 — Analyses of the transcript levels of AtPRPL18 in OE-1 and OE-2. [file Image_3.TIF]
